# Characterization of functional protein complexes from Alzheimer’s disease and healthy brain by mass spectrometry-based proteome analysis

**DOI:** 10.1038/s41598-021-93356-9

**Published:** 2021-07-06

**Authors:** Beena Hasan, Ayesha Khan, Christof Lenz, Abdul R. Asif, Nikhat Ahmed

**Affiliations:** 1 Neurochemistry Research Laboratory, Department of Biochemistry, University of Karachi, Karachi, 75270 Pakistan; 2 Institute of Clinical Chemistry, University Medical Center Göttingen, Göttingen, 37075 Germany; 3 Max Planck Institute for Biophysical Chemistry, Bioanalytical Mass Spectrometry Group, Göttingen, 37077 Germany

**Keywords:** Biochemistry, Biological techniques, Molecular biology, Neuroscience

## Abstract

Alzheimer’s disease (AD) is a complex neurodegenerative disorder with impaired protein activities. Proteins in the form of complexes have a ubiquitous role in diverse range of cellular functions. The key challenge is to identify novel disease associated protein complexes and their potential role in the progression of AD pathology. Protein complexes were obtained from AD brain prefrontal cortex and age matched controls by Blue Native-Polyacrylamide Gel Electrophoresis. A proteomic analysis was performed using second dimension SDS-PAGE followed by nano LC–MS/MS. Differentially expressed proteins were mapped to existing biological networks by Ingenuity Pathway Analysis (IPA). A total of 13 protein complexes with their interacting proteins were resolved on SDS-PAGE. We identified 34 protein spots and found significant abundance difference between the two experimental samples. IPA analysis revealed degeneration of neurons and cell death as a major consequence of protein dysregulation. Furthermore, focused network analysis suggested an integrated regulation of the identified proteins through APP and MAPT dependent mechanisms. The interacting differentially expressed proteins in AD were found to be part of concomitant signaling cascades terminating in neuronal cell death. The identified protein networks and pathways warrant further research to study their actual contribution to AD pathology.

## Introduction

Aggregates of intraneuronal neurofibrillary tangles (NFTs) and extracellular β-amyloid (Aβ) plaques are the hallmarks of Alzheimer’s disease (AD)^1^. Although their specific roles in etiology or pathology of AD have been extensively studied, the mechanism by which these structures in association with other molecular factors contribute to neuropathology remains elusive^2^. It is therefore important to decipher AD associated aggregates and their interactions with multiple proteins involved in cellular functioning and related metabolic pathways. Multiple proteomic strategies and systems biology approaches identified markers of AD onset and progression^3^.

Proteins accomplish their biological function in association with other proteins and rarely work in isolation. Often aberrant non-covalent interactions between protein components will result in disease states. The potential biological role of a protein in an orchestrated cellular environment can be inferred from its protein–protein interaction (PPI) network^4^. These interactions may be permanent or transient and can be altered by the conformational state of protein or its post translational modification (PTM). The data of human protein–protein interactions (PPIs) brought insights to the network biology of diseases and explained the inter-relationships among disease-related genes and proteins^5^.

Multiple proteins and their networks are altered in AD which results in altering the overall physiological state of the cell^1^. Proteomic alterations and network analysis can be used as powerful tools to study the complex neurodegenerative disorder of AD. Recent studies highlight the involvement of essential genes, proteins, signaling pathways and potential therapies against diseases using PPI dataset^6^, however interacting proteins and their networks in AD are still elusive.

Due to a lack of data on protein complexes, their interactions and implications on neurodegenerative diseases, this study was undertaken to explore the interacting components of protein complexes and their impairment subsequent to expression aberrations in AD. Our analysis underscores pivotal contribution of aberrantly expressed proteins in various molecular and biological processes chiefly autophagy leading to neuronal degeneration in AD. Moreover, our study also emphasizes the hampered role of metabolic enzymes in cytoskeleton destabilization, providing a better understanding of AD pathology.

## Methods

### Human brain tissue collection and characteristics

Autopsied brain samples of prefrontal cortex from pathologically confirmed well characterized Alzheimer’s disease patients and age matched unaffected controls were obtained with prior informed consent of the donor from MRC Sudden Death Brain Bank, The University of Edinburgh, UK. These samples were obtained from short postmortem interval autopsies of six control subjects and five AD patients with a mean age of 76 yrs. The tissues after excision were snap frozen in liquid nitrogen and stored at − 80 °C until further processed. The study has been designed in accordance with the guidelines for the use of human samples defined by ethical review board of University of Karachi, Karachi, Pakistan.

### Isolation and solubilization of protein complexes

The isolation of protein complexes from the human brain prefrontal cortical region was performed according to the method of Wittig et al.^7^ with slight modifications Briefly, the brain prefrontal cortex tissue (50 mg) from AD and age-matched control was homogenized in sucrose buffer (250 mM sucrose, 20 mM imidazole/HCl, pH 7) using pellet pestle motor hand homogenizer (10–20 strokes). The homogenized samples were centrifuged at 20,000*g* for 10 min to obtain nuclei, mitochondria and large cell fragments in the pellet. The pellet was further homogenized in solubilization buffer A (NaCl 50 mM, Imidazole 50 mM, 6-aminohexanoic acid 2 mM, EDTA 1 mM, pH 7). The protein complexes were then solubilized in 20% dodecyl-maltoside for 30 min with subsequent centrifugation at 100,000*g* for 15 min. 50% glycerol and 5% Coomassie Brilliant Blue G-250 (CBB G-250) were finally added to the centrifuged supernatant. Triplicates of each sample were used to ensure reproducibility.

### 2D BN/SDS-PAGE and image analysis

Blue-native PAGE was performed according to the method of Wittig et al.^7^ with some alterations for attaining reproducible results as described earlier^8^. Briefly, the protein complexes were separated on 4–16% blue-native gradient separation gel with a 3.5% stacking gel. Electrophoresis was carried out at 100 V (4 °C) with the cathode buffer (7.5 mM Imidazole, 50 mM Tricine) containing 0.02% (w/v) CBB G-250 and the anode buffer (25 mM Imidazole, pH 7.0). It was further continued at 200 V with 0.002% of CBB G-250 containing cathode buffer. BN gel was fixed (50% methanol, 10% acetic acid and 100 mM ammonium acetate) followed by CBB G-250 staining. During first dimension electrophoresis, a second-dimension gradient SDS-PAGE gel (7.5–12.5%) was pre-poured in BIO-RAD mini gel plates (1.5 mm spacers) leaving a gap of 1 cm above the gel. Lanes were cut from the first-dimension gel and then slid into place horizontally on top of the second-dimension gel using sample gel (3.5%) to seal the two gels. Single tooth of the comb was also adjusted during gel polymerization for marker. In second-dimension run was performed, initially at 100 V while in the sample gel and later continued in gradient gel at 15 mA for approximately 3 h. Proteins were visualized using standard CBB G-250 staining protocols^9^. The BN gel images of protein complexes were densitometrically analyzed by Quantity One software (BIO-RAD). Whereas, the SDS-PAGE resolved protein components were analyzed by Melanie 7.0 (GeneBio) according to published protocol^8^. The spots obtained from AD prefrontal cortex were compared with those of age matched controls.

### In-gel digestion and protein identification by nano LC–MS/MS analysis

Protein spots of interest were manually excised and prepared for in-gel digestion by dehydration followed by rehydration for digestion with 40 μL trypsin (10 ng/μL in 100 mM ammonium bicarbonate, pH 7.4; Promega, Mannheim, Germany) in ice for 45 min. After the removal of trypsin which was in excess digestion solution devoid of trypsin was added to make up the volume. Repeated addition of ACN and trifluoroacetic acid (TFA) in varying concentrations were used to extract the peptides following overnight digestion at 37 °C. At the end of the extraction, solutions were pooled, vacuum dried and re-dissolved in 0.1% TFA. The samples were separated on an analytical reversed phase-C18 column (0.075 mm ID × 200 mm, Reprosil-Pur 120 C18-AQ, 3 µm) after enrichment on a self-packed reversed phase-C18 precolumn (0.15 mm ID × 20 mm, Reprosil-Pur120 C18-AQ 5 µm) using a 15 min linear gradient of 5–35% acetonitrile/0.1% formic acid (v:v) at 300 nl min^-1^). The eluent was analyzed on a Q Exactive hybrid quadrupole/orbitrap mass spectrometer (Thermo Fisher Scientific, Dreieich, Germany) equipped with a Flex Ion nanoSpray source and operated under Xcalibur 2.4 software. Each experimental cycle was of one full MS scan across the 350–1600 m*/z* range acquired at a resolution setting of 70,000 Full-Width at Half-Maximum (FWHM), an Automatic Gain Control (AGC) target of 1 * 10e6 and a maximum fill time of 60 ms. Up to the 10 most abundant peptide precursors of charge states 2 to 5 above a 2 * 10e4 intensity threshold were then sequentially isolated at 2.0 FWHM isolation width, fragmented with nitrogen at a normalized collision energy setting of 25%, and the resulting product ion spectra recorded at a resolution setting of 35,000 FWHM, an AGC target of 2 * 10e5 and a maximum fill time of 120 ms. Selected precursor m/z values were then excluded for the subsequent 6 s^10^.

### Data processing

Protein identification was carried out using MASCOT 2.4 software (Matrix science, London, United Kingdom) identified against the UniProtKB *Homo sapiens* reference proteome. The database was searched using trypsin as enzyme and iodoacetamide as cysteine blocking agent. Methionine oxidation was set as a variable modification whereas up to two missed tryptic cleavages were allowed. Search tolerances were specified to 10 ppm for the precursor mass and 0.05 Da for-fragment masses, and ESI-QUAD-TOF as the instrument type^11^.

### Biocomputational analysis of identified proteins

PANTHER databases (Protein Analysis through Evolutionary Relationship, http://pantherdb.org/ ) and Uniprot (http://www.uniprot.org/) were used for spatial, temporal and functional classification of the identified proteins in AD and control brain. To further elucidate the potential biological mechanisms and the complexity of AD pathology in terms of protein complexes their components and PPI involving the identified proteins in the AD brain, Ingenuity Pathway Analysis (IPA) algorithm (http://www.ingenuity.com) was used. Based on the identified interactions between proteins, IPA defines protein networks and characterizes common functional and canonical pathways, thereby offering additional information about the complex interactive links between the interacting proteins in the diseased brain.

### Co-immunoprecipitation and western blot analysis

In order to validate interaction of cytoskeletal protein (actin cytoplasmic; ACTB) with metabolic enzyme (Glyceraldehyde-3-PO4 dehydrogenase; GAPDH) co-immunoprecipitation was carried out. For precleaning of the lysate, protein Sepharose G beads were washed (× 4) with HEPES buffer at 2000–3000 rpm and mixed with the extracted protein sample at 4 °C for 30 min with continuous shaking. The sample was incubated with 5 μl anti-beta Actin antibody (mouse monoclonal [mAbcam 8226] to beta Actin antibody, ABCAM, UK) overnight following 4 °C centrifugation at 13,000 rpm for 5 min. Activated beads were then added and incubated for 4 h at 4 °C, washed with HEPES buffer and subjected to SDS-PAGE at 100 V for approximately 50 min. The electrophoretically resolved proteins were transferred to polyvinylidene fluoride membrane (PVDF; Amersham GE Healthcare) at 300 V for 3 h. The transferred membrane was blocked with 5% BSA for 1 h and then incubated with anti-GAPDH antibody (mouse monoclonal [mAbcam 9484] to GAPDH, ABCAM, UK) (1:2000), overnight. Tris-buffered saline with Tween (TBST) washing was carried out followed by secondary antibody (horse-radish peroxidase (HRP)-conjugated) incubation and color development using 3,3′,5,5′-Tetramethylbenzidine (TMB) reagent.

### Ethical approval

The study has been designed in accordance with the guidelines for the use of human samples defined by ethical review board of University of Karachi, Karachi, Pakistan.

## Results

### Expression of protein complexes in AD prefrontal cortex

**Figure 1:**
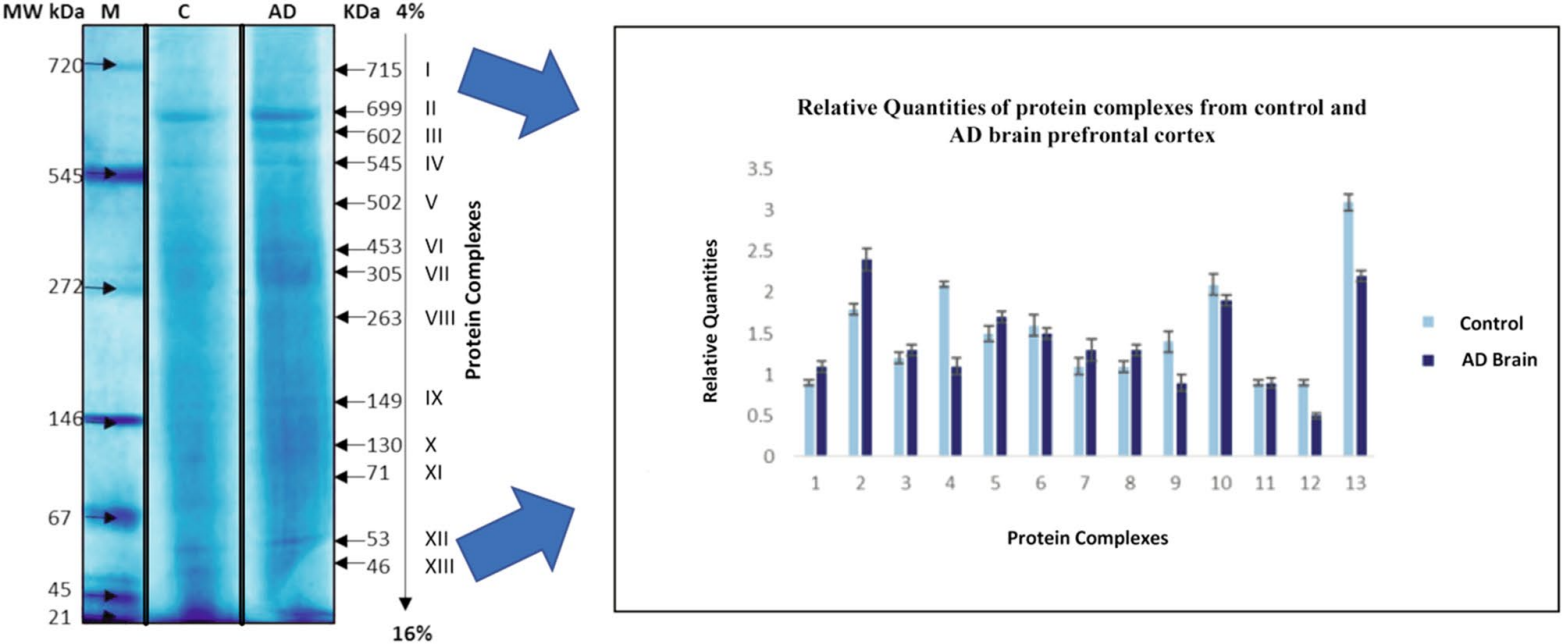
Pattern of protein complexes (120 µg) obtained from the separation of control and AD sample (lane 2 and 3) resolved on 1D BN-PAGE 4–16% gel. Complex IV and XIII represent significant expression alterations among ageing control and AD brain samples. M: Molecular weight marker; C: Healthy control subjects; AD: Alzheimer’s disease subjects.

In total, thirteen protein complexes (I-XIII) were detected on Blue Native-Polyacrylamide Gel Electrophoresis (BN-PAGE) with molecular masses ranging from 46 to 715 kDa (Fig. 1) (Suppl Fig. S1–S3). The individual complexes were comprised of molecular weights 715 kDa, 699 kDa, 602 kDa, 545 kDa, 502 kDa, 453 kDa, 305 kDa, 263 kDa, 149 kDa, 130 kDa, 71 kDa, 53 kDa and 46 kDa respectively. Significant decrease was observed in the expression level of complex IV comprised of immunoglobulin super family member 8 (IGSF8) and microsomal glutathione S transferase (MGST1) along with complex XIII comprised syntaxin-1A (STX1A) and tubulin polymerization promoting protein (TPPP) in AD brain as compared to control samples.

### Differentially expressed components of protein complexes

**Figure 2:**
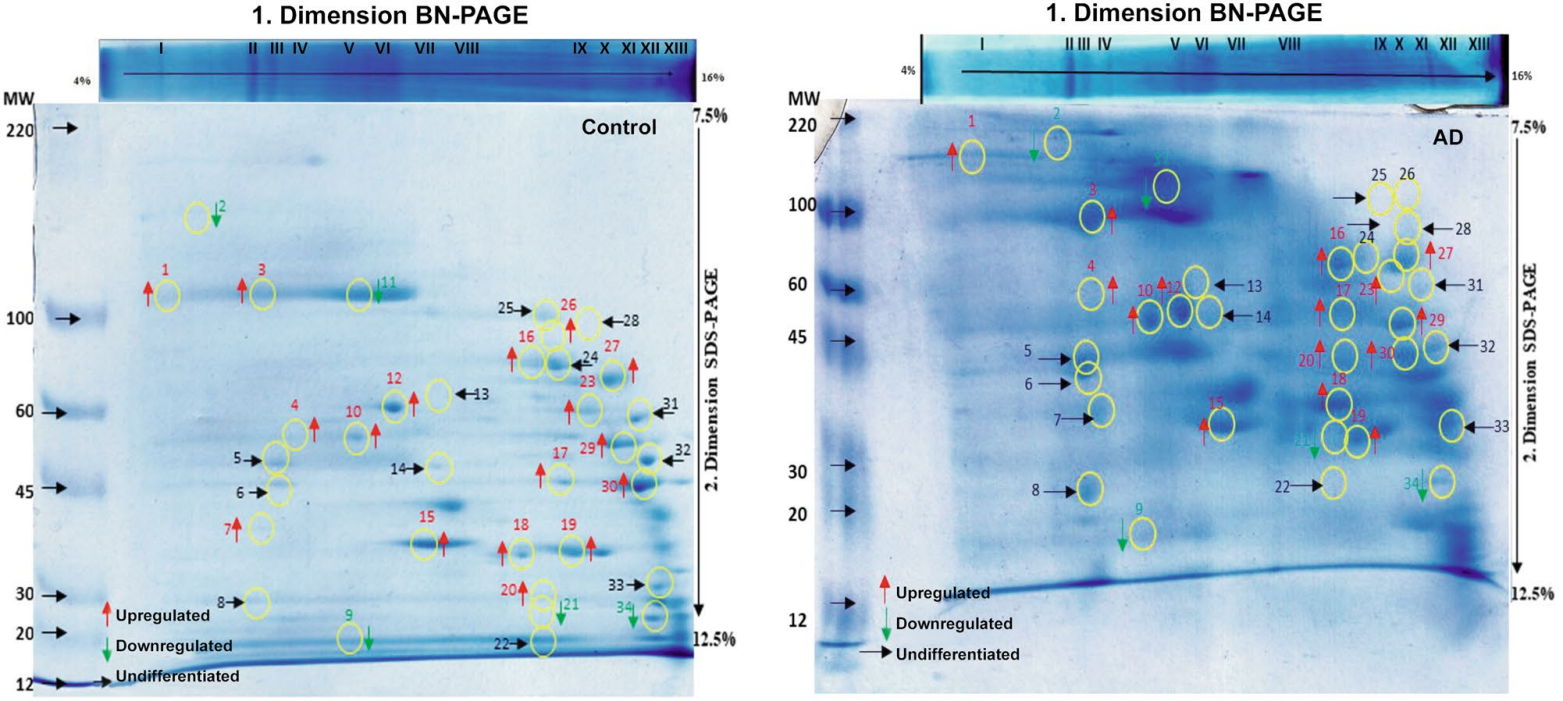
BN/SDS-PAGE map acquired from separation of ageing control and AD protein complexes isolated from human brain prefrontal cortex. Representative spots separated on 12.5–7.5% SDS-PAGE in second dimension and Coomassie stained. The identified spots with increased expression are marked in red, whereas lower expressed spots are marked in green. Undifferentiated protein spots are marked in black.

A total of 117 protein spots were detected, when the aggregates of BN-PAGE derived protein complexes were resolved on 12.5–7.5% SDS-PAGE. The image analysis revealed 96 statistically significant (*p* ≤ 0.05) differentially expressed protein spots out of which 33 protein spots showed increased expression, while 63 protein spots showed remarkable decrease expression in AD in comparison with control samples. The visibly prominent spots from 2D BN-PAGE gels pertinent to protein complexes were selected that appeared in all the replicate gels of control and AD samples with same shape and size on nearly the same position and subjected to mass spectrometric analysis (Fig. 2). The replicates provided in Figure S4–S8 in the supplementary material. A decreased expression of five components was observed in AD whereas seventeen protein components showed increased expression in AD compared with control samples. Although more than one protein was identified for a single spot (more than 5) the protein components were selected on the basis of their mascot score and maximum peptide matches along with sequences and also on the basis of their molecular weight and their position on gel. However, twelve expressed protein spots did not show significant differential expression. The detailed information of the identified proteins including the accession no, score, peptide matches, biological process, and subcellular localization with respective fold change is provided in supplementary material (Supplementary Table 1).

**Figure 3:**
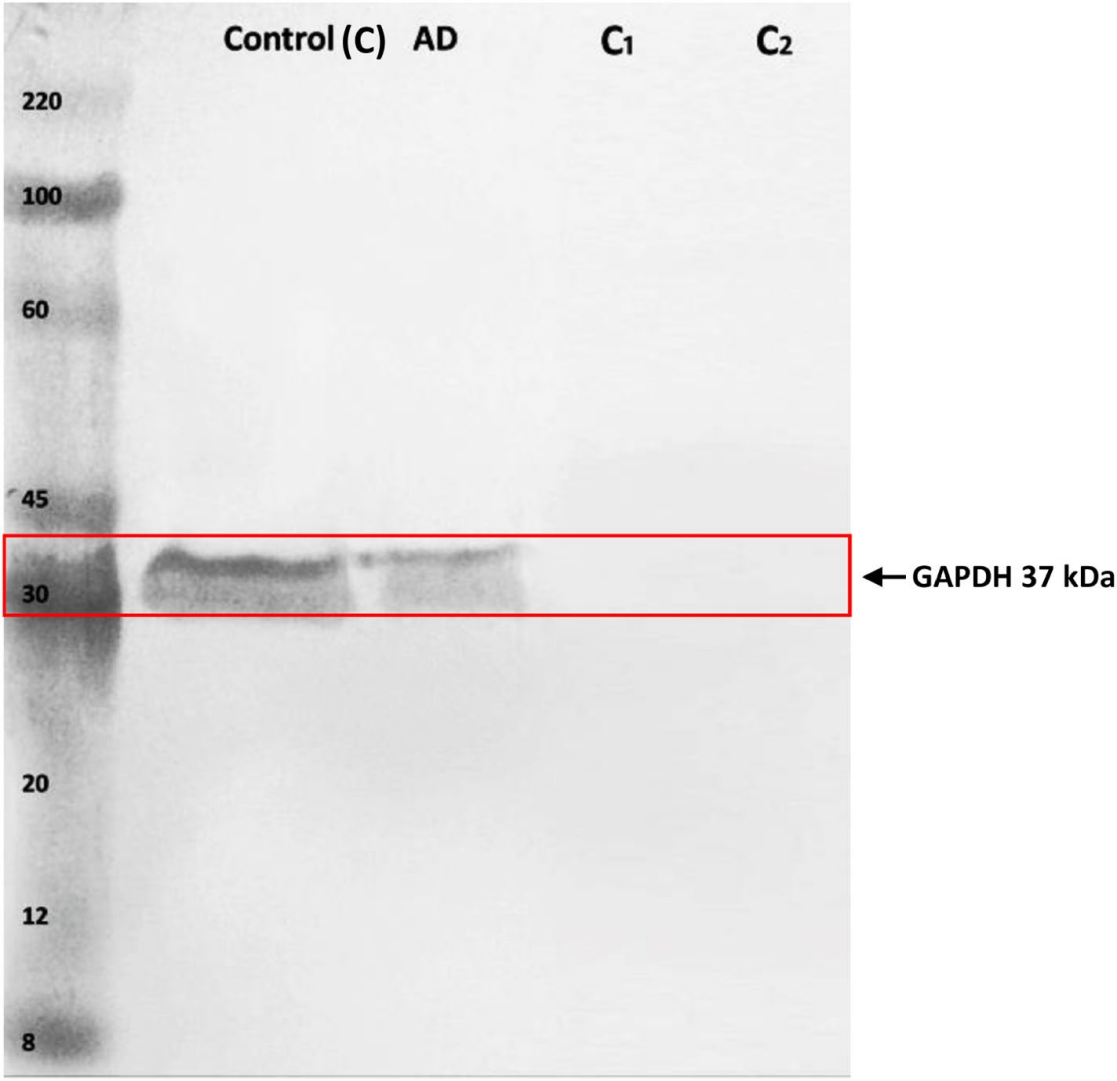
Co-immunoprecipitation of beta-actin with GAPDH for the identification of protein interaction in human brain prefrontal cortex. Human brain tissue proteins from AD patients and age matched control after solubilization in dodecyl-maltoside were collected using specific anti beta-actin antibody. The immunoprecipitaes were separated by SDS-PAGE electrophoresis followed by western blotting with anti GAPDH antibody. Immunoreactive band of GAPDH observed at 37 kDa. Representative gel obtained from triplicate set of experiments. Two controls were included in the co-IP analysis: (C1) protein sepharose beads were incubated with extracted proteins in the absence of anti beta-actin antibody, (C2) protein sepharose beads were incubated with anti beta-actin antibody in the absence of extracted proteins. C: control subjects; AD: Alzheimer’s disease patients; C1: control 1; C2: control 2.

One of the conventional interactions between GAPDH and ACTB obtained in complex VII was validated in control and AD samples (Fig. 3). A strong interaction between the two proteins observed in both the samples indicates that their altered expression did not impair the interactive binding of GAPDH and ACTB in AD (Supplementary Figure S9 and S10).

### Functional classification of the modulated proteins

**Figure 4:**
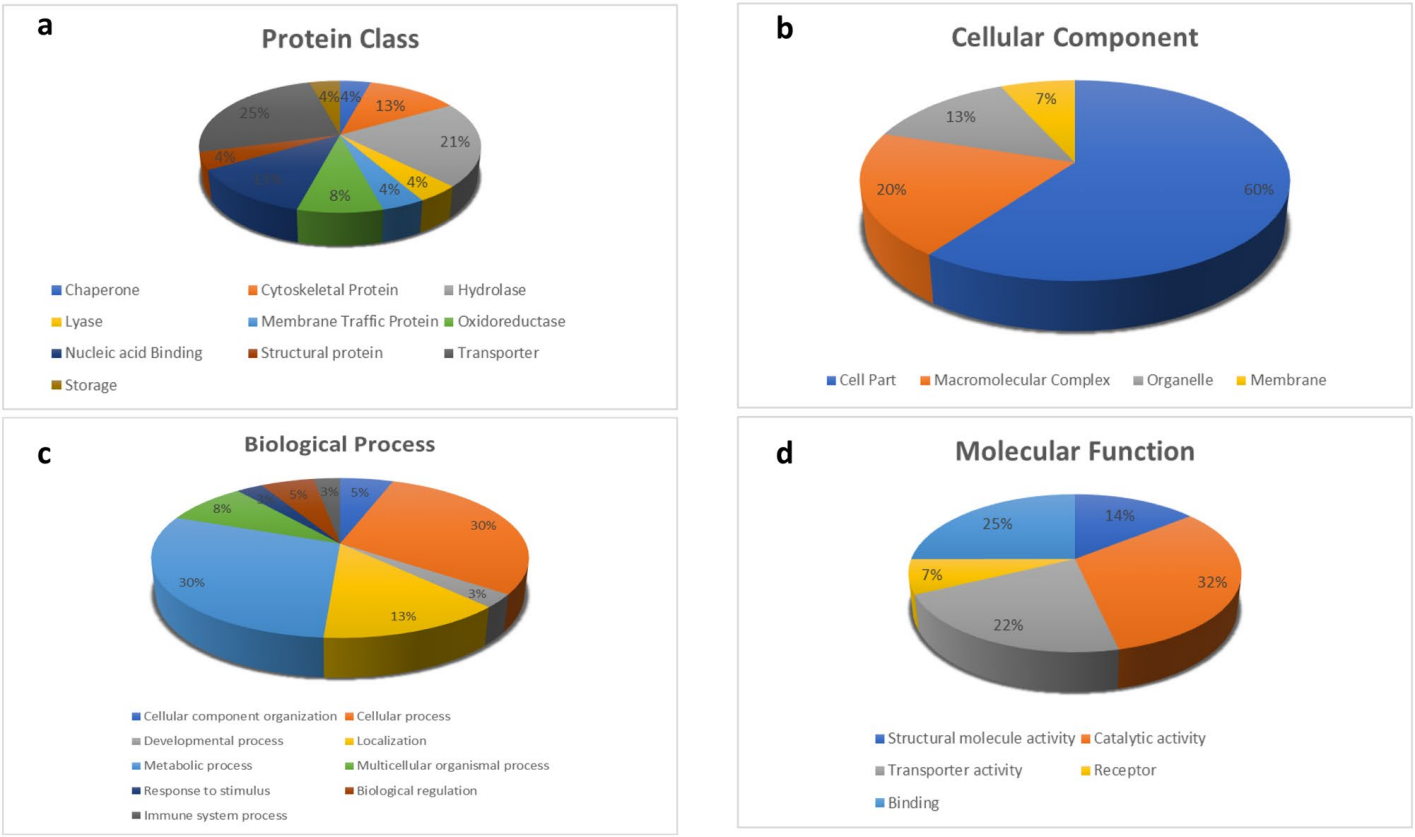
Functional classification of differentially expressed protein components of the 13 protein complexes in the prefrontal cortex of Alzheimer’s disease brain. The nano LC–MS/MS identified cortical proteins were characterized within the molecular function gene ontology (GO) category. Subcellular and functional categories were based on the annotations of GO using the online tool at www.pantherdb.org in the following categories (**a**) protein class, (**b**) cellular component, (**c**) biological process and (**d**) molecular function .

The identified differentially expressed proteins from prefrontal cortex were placed into broad functional categories by using PANTHER database (Fig. 4). Based on their function most of the proteins were associated with transporter role (25%) and enzyme hydrolases (21%). These proteins mainly constitute the cell part (60%) besides forming macromolecular assemblies (20%). The most populated gene ontology (GO) biological processes were found to be cellular and metabolic processes (30% each) along with localization (13%). Moreover, the three of the top five GO molecular functions enriched were: catalytic (32%), binding (25%) and transporter activity (22%). Collectively, most of the interacting proteins belong to the catalytic activity enriched in cellular and metabolic processes occurring in brain.

### Canonical pathway and protein functional network analysis

**Figure 5:**
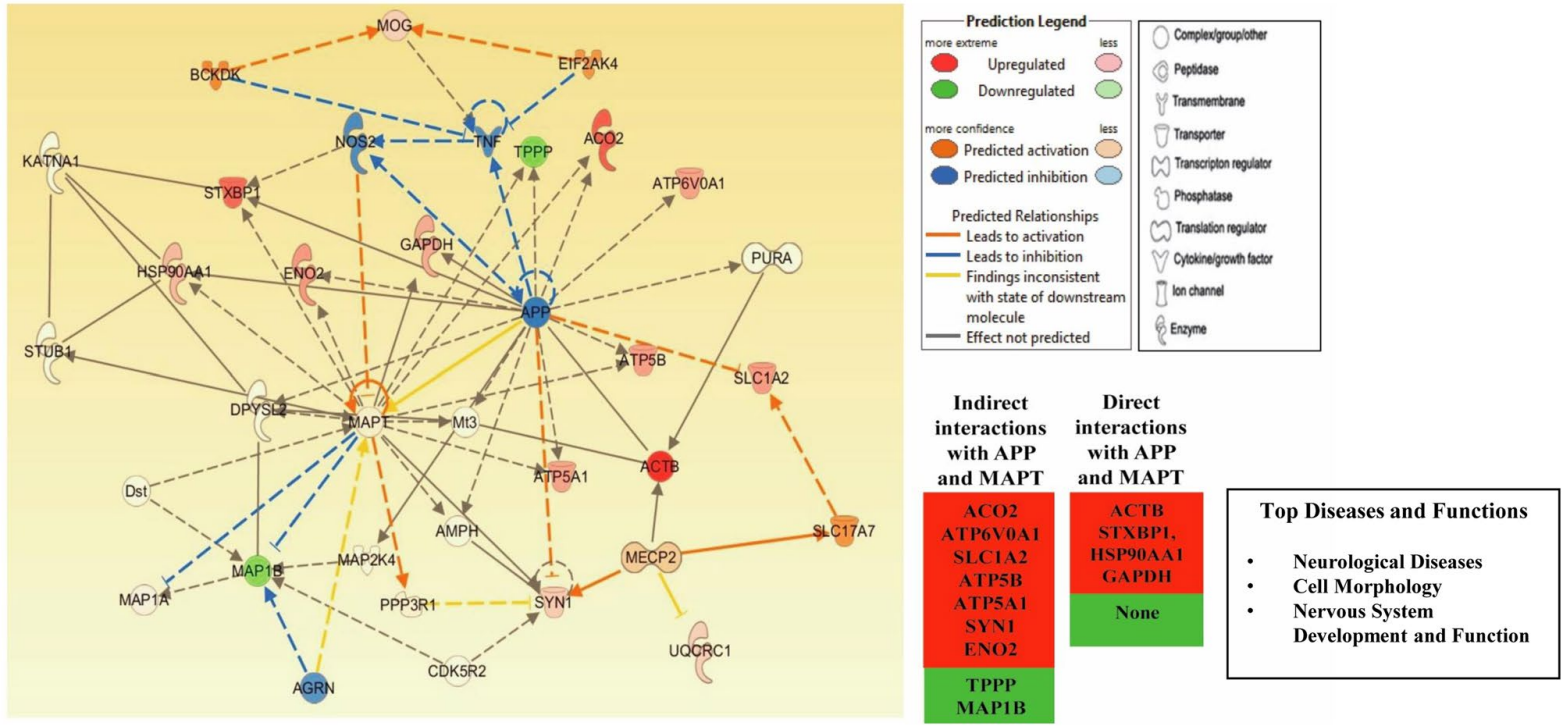
IPA based protein network involved in the development of neurological disorder altering cell morphology and function implicated in AD cortex. The IPA generate networks of differentially expressed proteins and their potential association with other known proteins in AD. The color of proteins indicates their up (red) and down regulated expression in AD in the current dataset, whereas the shapes of the proteins implies their molecular classes as outlined in the legend. Solid lines indicate direct interaction whereas dashed lines correspond to indirect relationship among the interacting proteins. The arrows indicate modulatory effect of a protein on its interacting proteins. APP and MAPT are central to the network creating hubs, interacting with most of the network proteins particularly the focus proteins in the original dataset and regulating them either directly or indirectly.

The Ingenuity Pathway Analysis (IPA) (QIAGEN) tool was used to search for top biological functions, pathways and protein networks that are affected in AD utilizing dataset of all dysregulated proteins based on propriety algorithm. The main representative molecular and cellular functions associated with our protein components include cell death and survival (*p* value 4.60E-02–3.88E-05), cellular assembly and organization, cell to cell signaling and interaction (*p* value 3.67E-02–3.17E-04) and molecular transport (*p* value 2.66E-02–1.72E-04). The impaired molecular and cellular functions consequently affect the functioning of canonical pathways including mitochondrial activity (*p* value 8.05 E-6) with defective oxidative phosphorylation (*p* value 2.48 E-5), glycolysis (*p* value 6.18 E-4) and gluconeogenesis (*p* value 6.18 E-4) disturbing the overall metabolism. One of the high-ranking pathways illustrates multidirectional interaction network of 35 differentially expressed AD proteins including 15 focus molecules; found in the network (Fig. 5).

**Figure 6:**
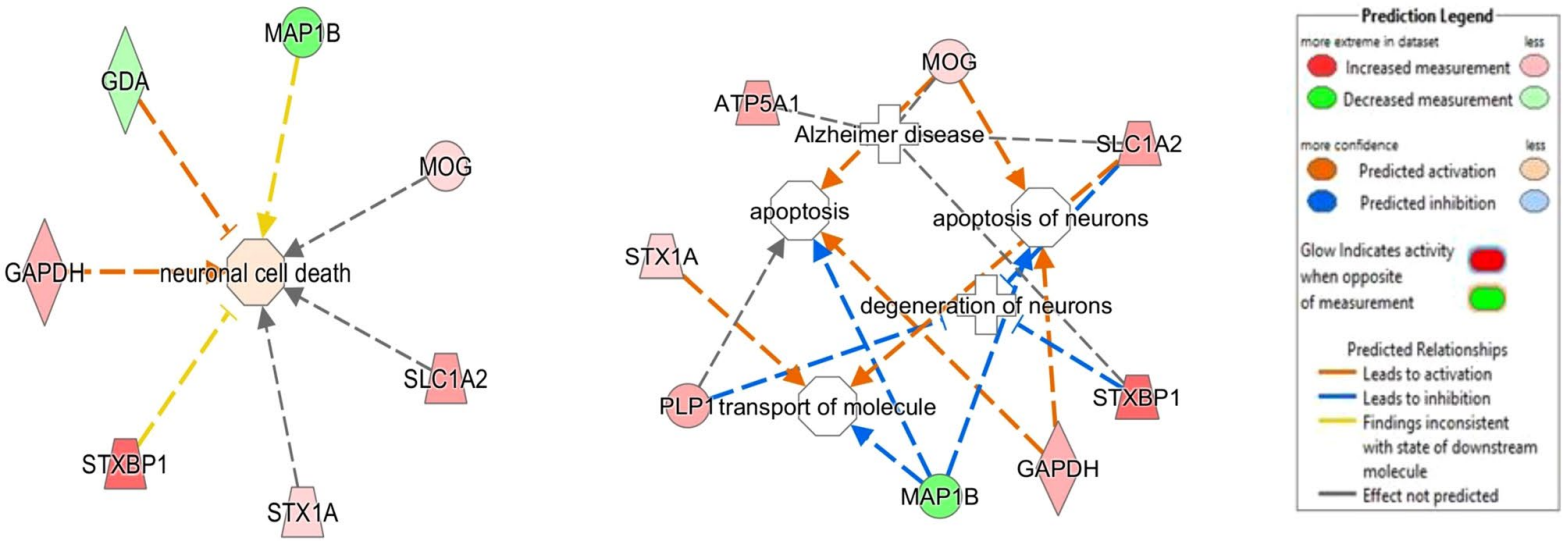
Diseases and functions associated with differentially expressed proteins implicated in AD prefrontal cortex. IPA constructs identified relationships between the differentially expressed proteins and associated disorders. Neuronal degeneration and cell death, apoptosis and transport of molecules are the chiefly disturbed pathways in Alzheimer’s disease.

This network implicates several protein nodes directly or indirectly correlated with our identified proteins. Interestingly, the interacting proteins of the network are regulated by amyloid precursor protein (APP) and MAPT (microtubule associated protein tau), creating interactive hubs associated with neurological symptoms, alteration in cell morphology and linked to nervous system development and function. IPA analysis further reveals the contribution of differentially expressed interacting proteins of AD in neuronal degeneration, cell death, apoptosis and transport of molecule (Fig. 6).

## Discussion

This study provides an analysis of cellular protein complexes and their novel binding partners in the cortical region of the brain together with its implications in AD pathology. BN-PAGE is frequently used to detect the binding partners in protein/protein complexes. Here, it elucidated the significant differential expression of protein complexes (I–XIII) between AD and normal healthy control cortical samples. The aberrations in protein complexes become evident in the second dimension SDS-PAGE when resolved in to their binding counterparts which show differential expression of several protein components of each complex (Fig. 2, Supplementary Table 1) followed by their identification through Nano LC–MS/MS analysis.

Some of the notable protein components with prominent differential expression includes FRIH with increased expression in AD, implicated in apoptosis and various neurodegenerative diseases^12^ and correlates well with increased iron load in AD. This contributes to a compensatory mechanism for increased oxidative stress, generated by Aβ aggregates in the presence of iron^13^. Transporters being one of the major protein class found in this study with differential expression includes SLC4A4. It has been hypothesized that decrease expression of SLC4A4 results in respiratory alkalosis and hypoxic condition counteracted by pH decline and decrease in neuronal excitability, the conditions which are commonly observed in AD^14^. Another transport protein ATP6V0A1 is critical for decreasing the intralumenal pH during autophagic vacuolar (AV) clearance for optimal protease activation^15^. A new mechanistic finding of this study is increased expression of ATP6V0A1 which decreases the pH in response to increased autophagosome production in AD. However, there is impaired autophagosome clearance due to decrease expression of MAP1B as observed in our study. MAP1B is crucial in microtubule bundling^16^ and is required for efficient transport of autophagosomes^17^. The increased expression of ATP6V0A1 with decrease MAP1B suggests disturbed cellular proteostasis exacerbating AD pathology. Additionally EAAT2 an upregulated transporter protein of the same complex in AD brain has been implicated in regulating the excitotoxic concentration of synaptic glutamate which promote hyperphosphorylated tau and tangle formation^18^. A significant decline in MGST1can modulate the signaling pathways controlling apoptosis, cell proliferation, and differentiation, consequently; leading to elevated oxidative stress in brain with decreased antioxidant species^19^.

Another protein HSP90A which stimulate the inflammatory pathways in AD, in turn affect the Aβ and tau pathologies^20^. Elevated expression of HSP90A can be correlated with the aberrant aggregation and accumulation of tau and Aβ toxicity along with an increased cytokine production. It also acts as a co-chaperone of HSP70 in chaperone mediated autophagy (CMA). A direct crosstalk links chaperone mediated autophagy and macroautophagy whereas blockage of the later leads to the up-regulation of CMA^21^. Impeding macroautophagy is also predicted by the decrease in MAP1B and elevated VPP1 expression ensuing in overexpressed HSP90A accelerating chaperone mediated autophagy. The implication that neurodegenerative associated proteins with diverse role in cellular systems particularly the HSP90A, MAP1B, VPP1, govern the process of autophagy is not surprising. Although not associated with autophagy, down regulation of ACTN1 is found to be associated with apoptosis as it appears to be implicated in the organization of cytoskeleton by actin bundling^22^.

Since GAPDH, ACTB and MAP1B are the components of a single complex, we suggest that GAPDH is involved in the regulation of crosstalk between microtubules and actin microfilaments through MAP1B. Moreover GAPDH is suggested to trigger apoptosis following cytotoxicity^23^ due to its interaction of with the β-amyloid precursor protein (β-APP), the β-amyloid protein (β-AP)^23^ and tau^24^. The increased expression of GAPDH can be associated with an increased aggregation and accumulation of tau and Aβ in intracellular and extracellular regions. MAP1B, GFAP and PLP are postulated to modulate the cytoskeleton structure and dynamics leading to progression of cell death in AD which has not been previously emphasized.

We are reporting for the first time an important observation depicting coalition of HSP90B and STXB1 predicting the role of HSP90B in exocytosis via SNARE complex. Although HSP70 has earlier been identified to interact with syntaxin and regulating exocytosis ^25^ but HSP90B has never been detected with syntaxin and syntaxin binding protein. STXB1identified with elevated expression, is found exclusively in presynaptic nerve terminals and binds to syntaxin-1 preventing it from interacting with docking fusion proteins and exocytosis. This is reversed by STXB1 phosphorylation and has been postulated to increase neurotransmission and may play a role in APP processing and Aβ production^26^.

TPPP is primarily expressed in oligodendrocytes and modulates the dynamics and stability of the microtubular network besides binding to Aβ^27^. Our study has reported the significant decrease in the expression of TPPP in AD brain. This altered expression along with its perturbed modification by ERK-2 pathway might provoke the disassembly of microtubules^28^. The other protein of this complex STX1A is a component of SNARE complex at the presynaptic membrane involved in exocytosis^29^. Aβ oligomers formed intracellularly during AD binds directly with STX1A hampering SNARE mediated exocytosis leading to cognitive decline^30^. Earlier studies have reported decreased expression of STX1A observed in AD brain impeding synaptic function^31^. However, no significant alteration in STX1A has been observed as a component of protein complexes in the present study. The interaction between TPPP and STX1A seems indirect as TPPP binds with tubulin for the assembly of microtubules whereas STX1A binds with tubulin for anchorage. The decrease expression of TPPP suggests impaired STX1A trafficking towards synapse.

The study implicates that cell morphology is disturbed accompanied with disturbance in neurological functioning as a result of alteration in proteins interacting with each other in the form of network with APP and MAPT forming strong hubs. Four of the focal proteins are in direct contact whereas nine proteins are indirectly associated with APP and MAPT. Since these two proteins are the hall marks of AD, their interaction with differentially expressed proteins suggests their role in developing and devastating the symptoms of AD. The differential expression of proteins along with altered post translational modifications leads to degeneration of neurons hampering transport mechanism, impeding cell viability, disturbing cellular proteostasis, leading to neuronal apoptosis and impaired motor functions.

Here it is pertinent to mention the limitations of the different techniques available to study the native protein complexes involving the acceptance of limits imposed by centering on the interacting triangle of sensitivity, specificity and throughput.

## Conclusions

The differential expression of protein components interacting in the form of complexes may be the initiation point of the disease. Over time the autophagic pathway, vesicle docking, and cytoskeletal destabilization may become overwhelmed, which in turn facilitates the propagation of disease associated aggregates throughout neuron and glial cells. However, the exact mechanism of initiation of neurodegenerative process and how the protein expression and interaction terminate in cell death needs further investigation. Moreover, quantifying complexes in AD will be useful for further understanding of onset and progression of AD and its associated consequences.

## Supplementary Information

Supplementary Figure S1.

Supplementary Figure S2.

Supplementary Figure S3.

Supplementary Figure S4.

Supplementary Figure S5.

Supplementary Figure S6.

Supplementary Figure S7.

Supplementary Figure S8.

Supplementary Figure S9.

Supplementary Figure S10.

Supplementary Legends.

Supplementary Table 1.
