# Supplementary figures and images for: Characterization of functional protein complexes from Alzheimer’s disease and healthy brain by mass spectrometry-based proteome analysis
Source: Sci Rep. 2021 Jul 6;11:13891. doi: 10.1038/s41598-021-93356-9 (PMC8260596; doi:10.1038/s41598-021-93356-9)

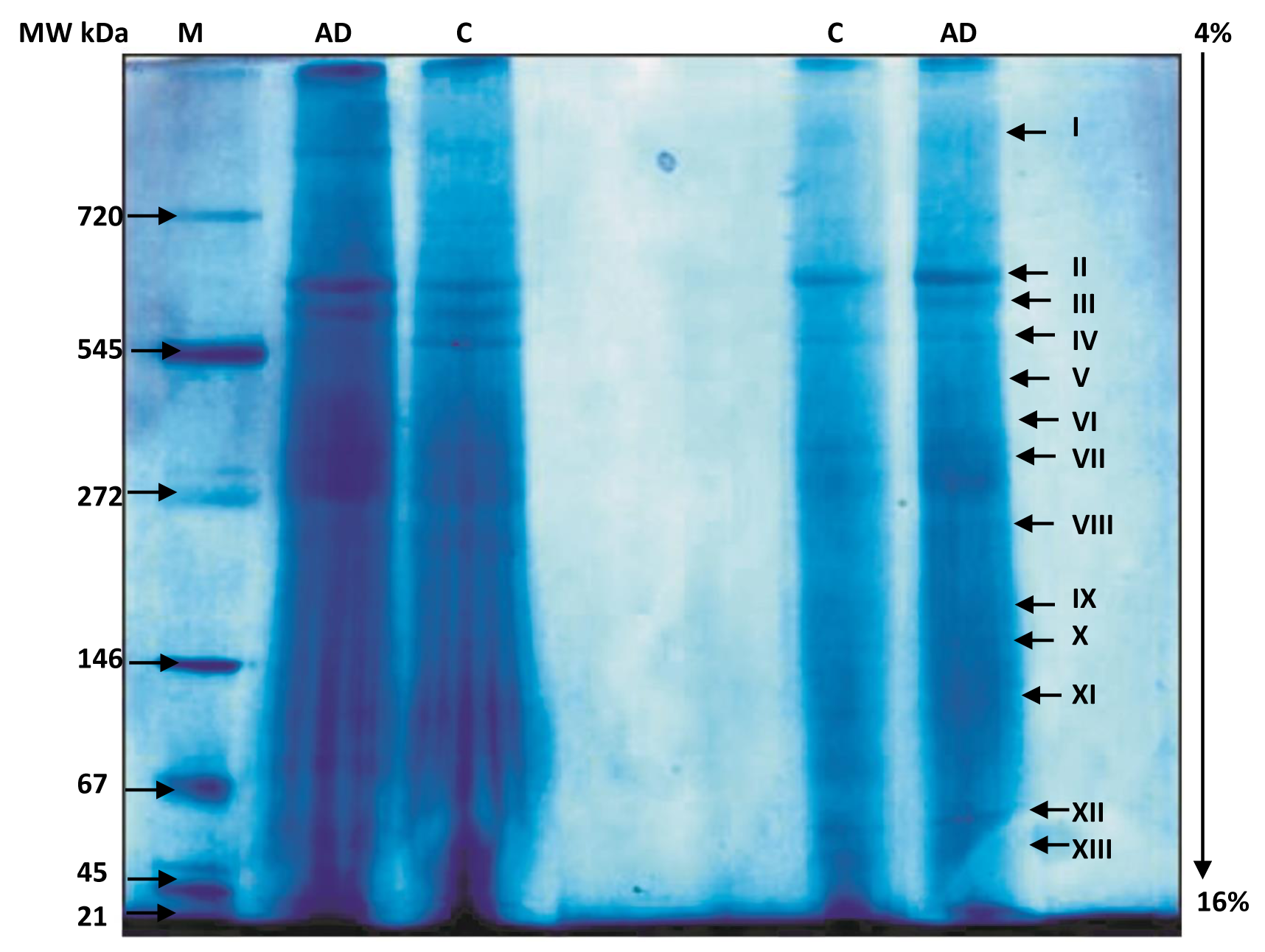

Supplement: Supplementary file 1 — Supplementary Figure S1. [file 41598_2021_93356_MOESM1_ESM.tif]

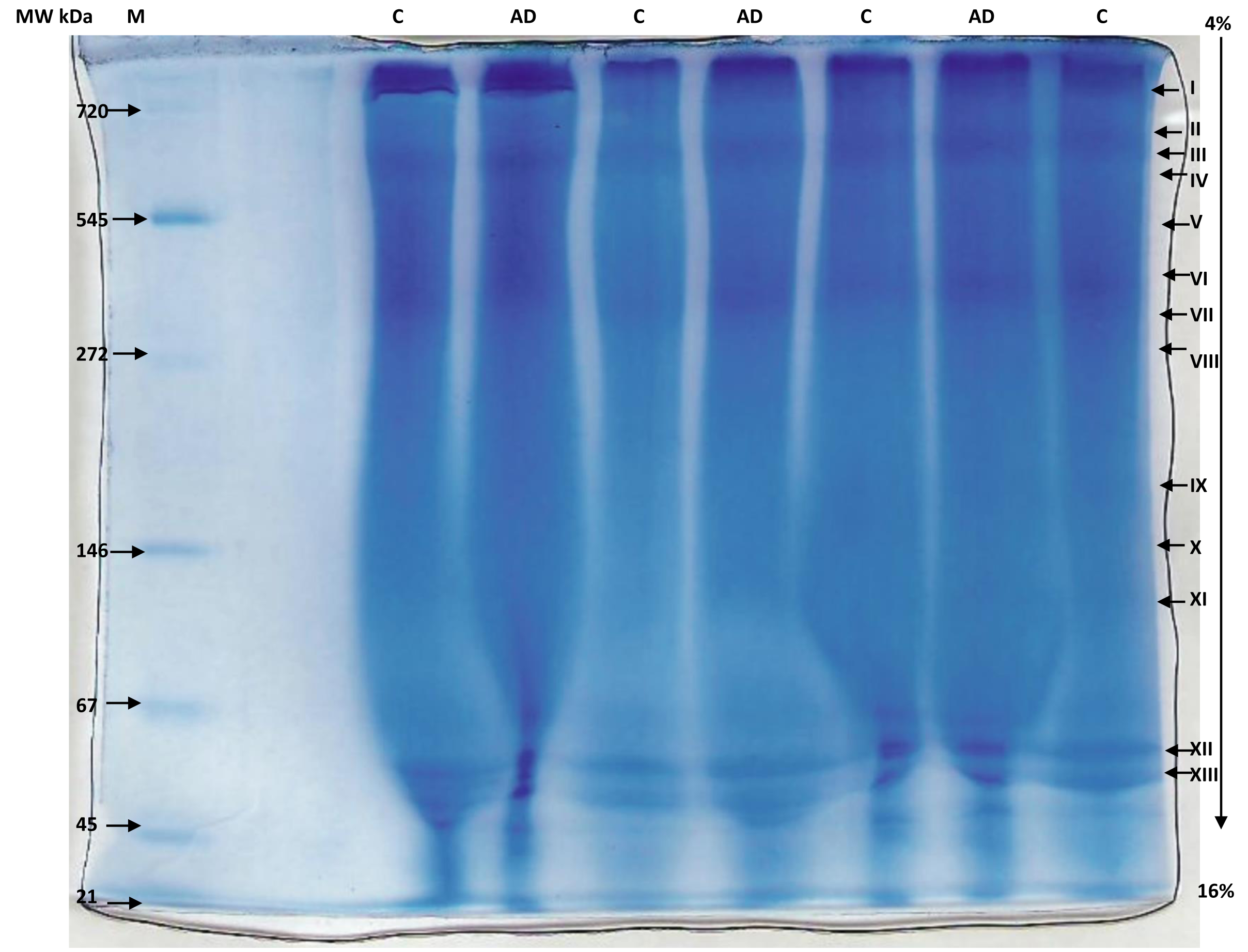

Supplement: Supplementary file 2 — Supplementary Figure S2. [file 41598_2021_93356_MOESM2_ESM.tif]

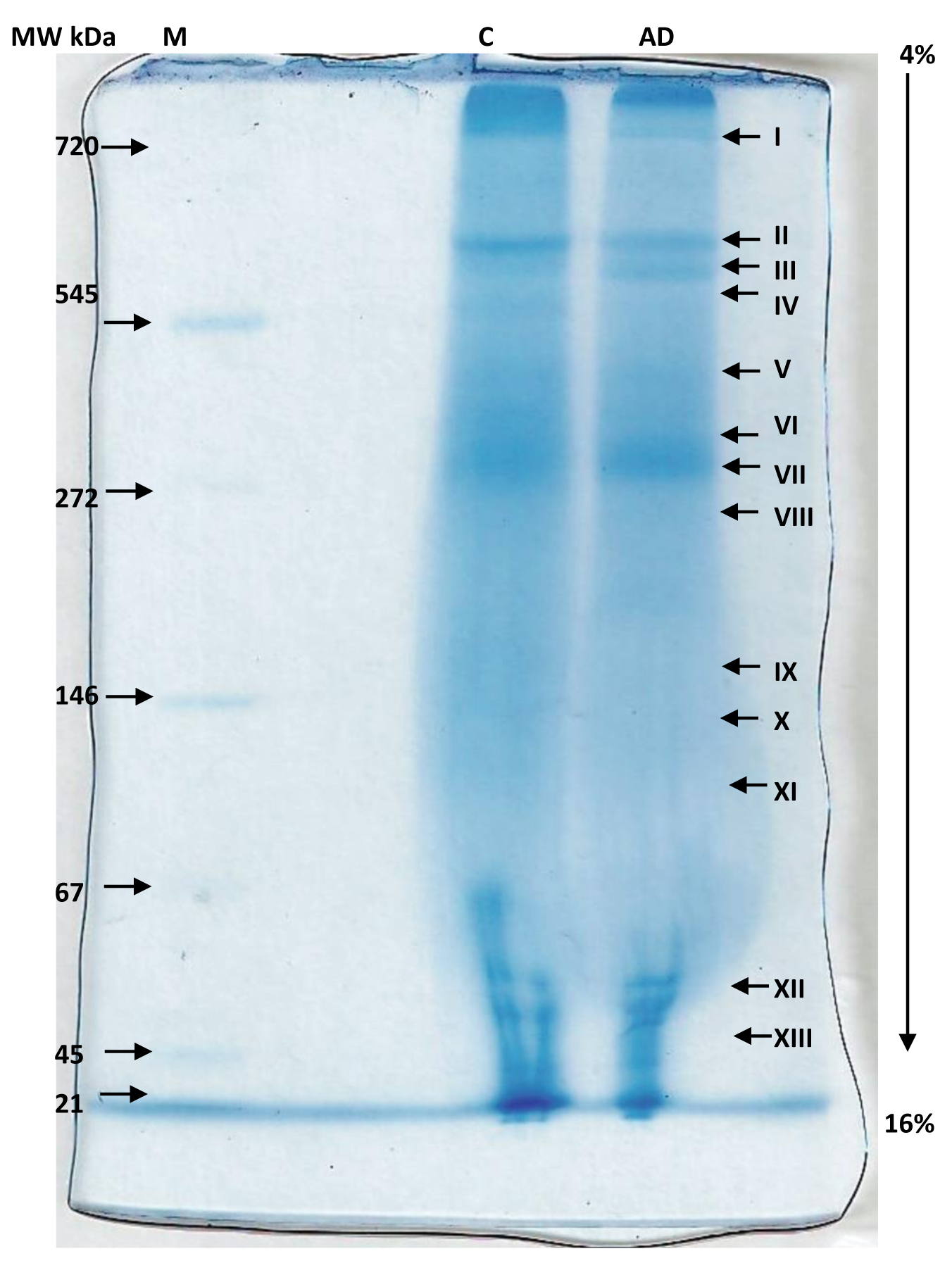

Supplement: Supplementary file 3 — Supplementary Figure S3. [file 41598_2021_93356_MOESM3_ESM.tif]

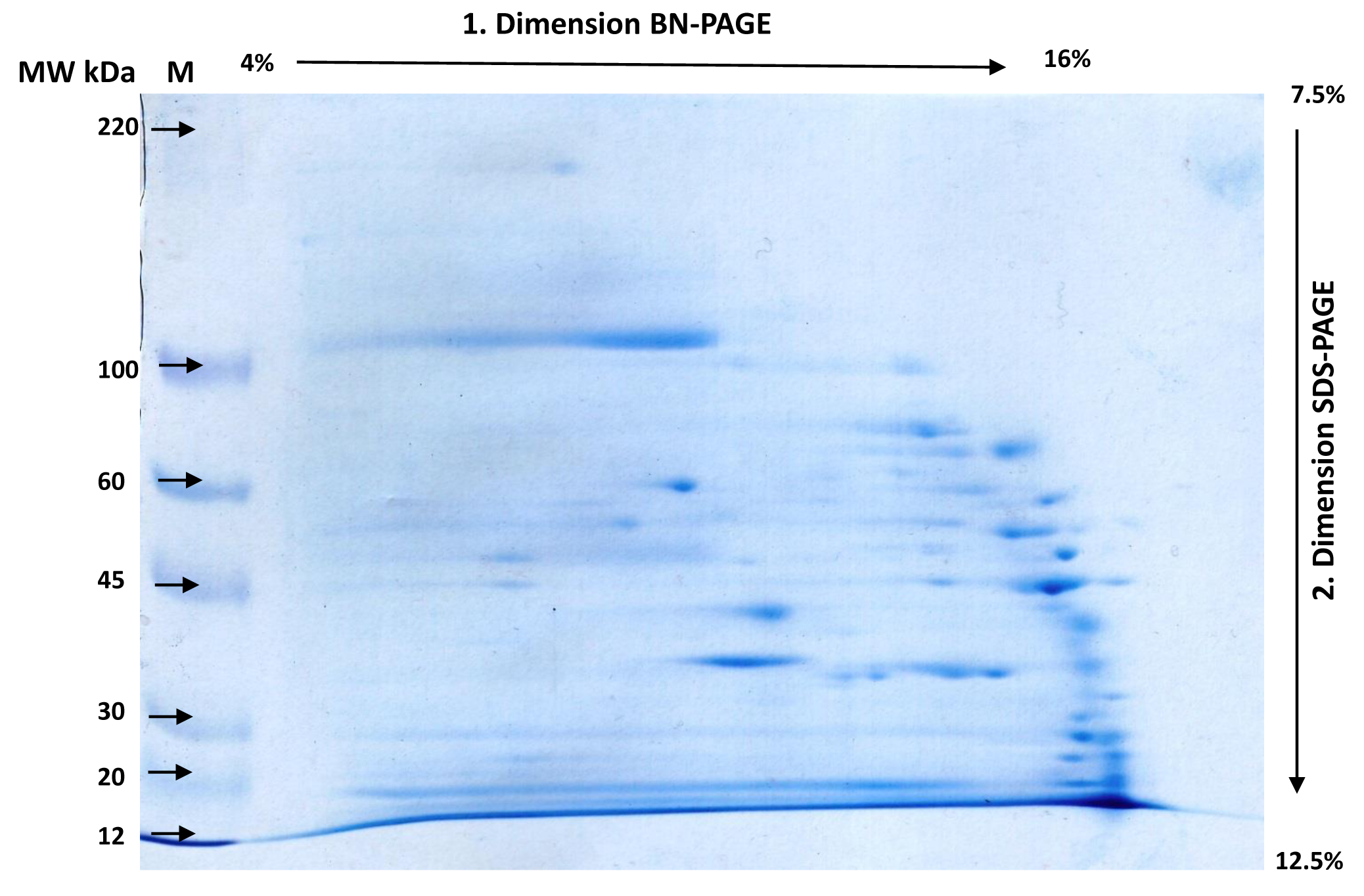

Supplement: Supplementary file 4 — Supplementary Figure S4. [file 41598_2021_93356_MOESM4_ESM.tif]

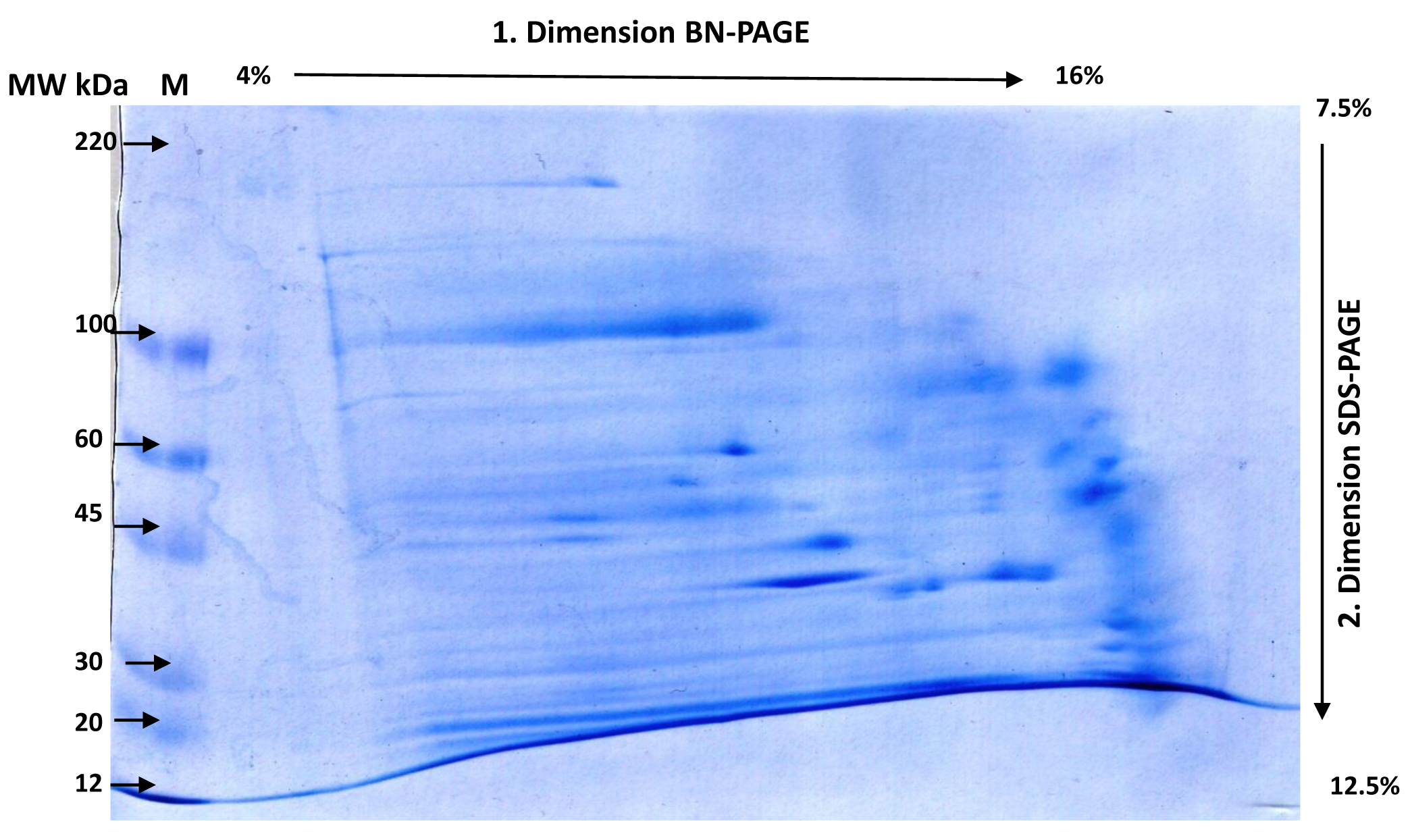

Supplement: Supplementary file 5 — Supplementary Figure S5. [file 41598_2021_93356_MOESM5_ESM.tif]

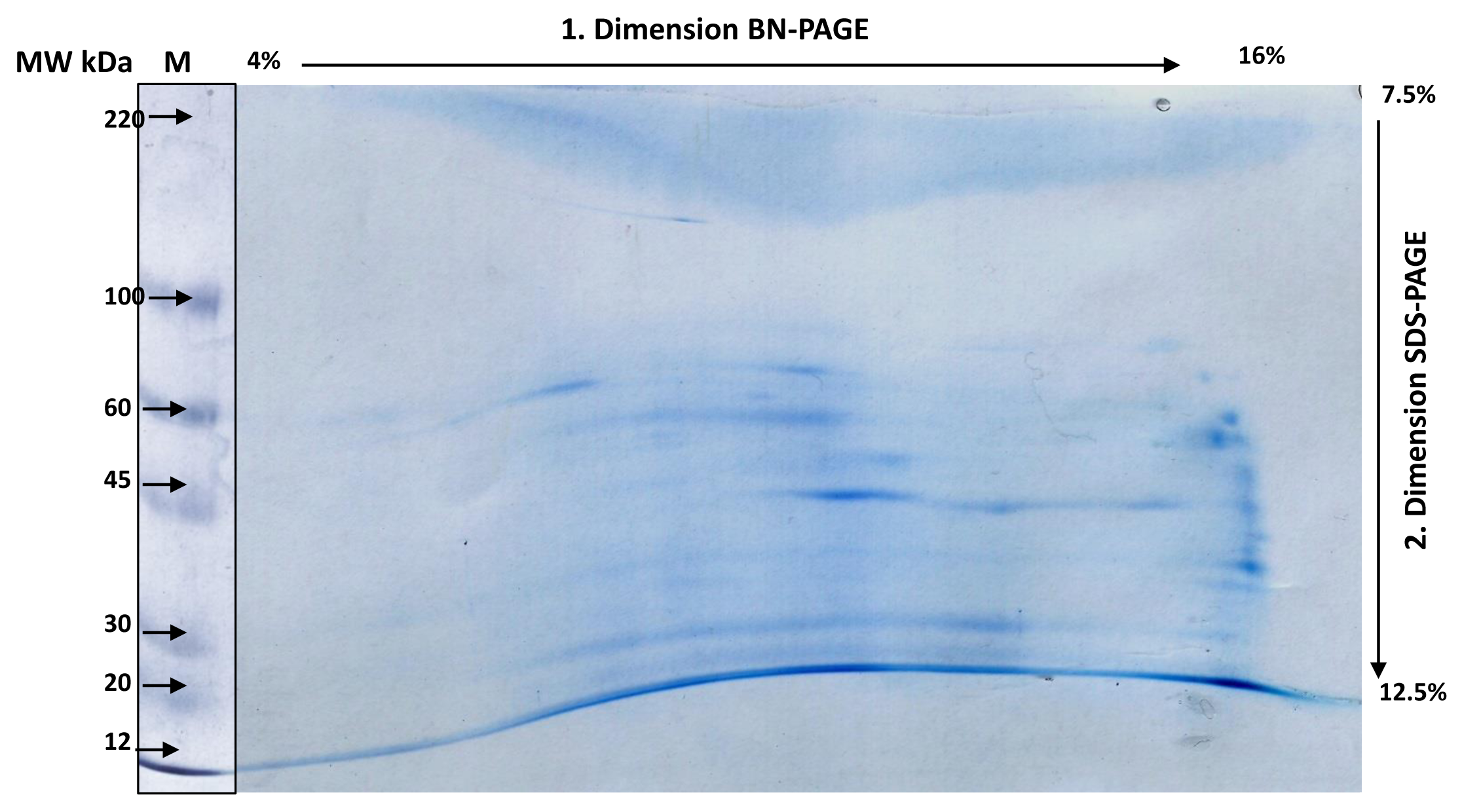

Supplement: Supplementary file 6 — Supplementary Figure S6. [file 41598_2021_93356_MOESM6_ESM.tif]

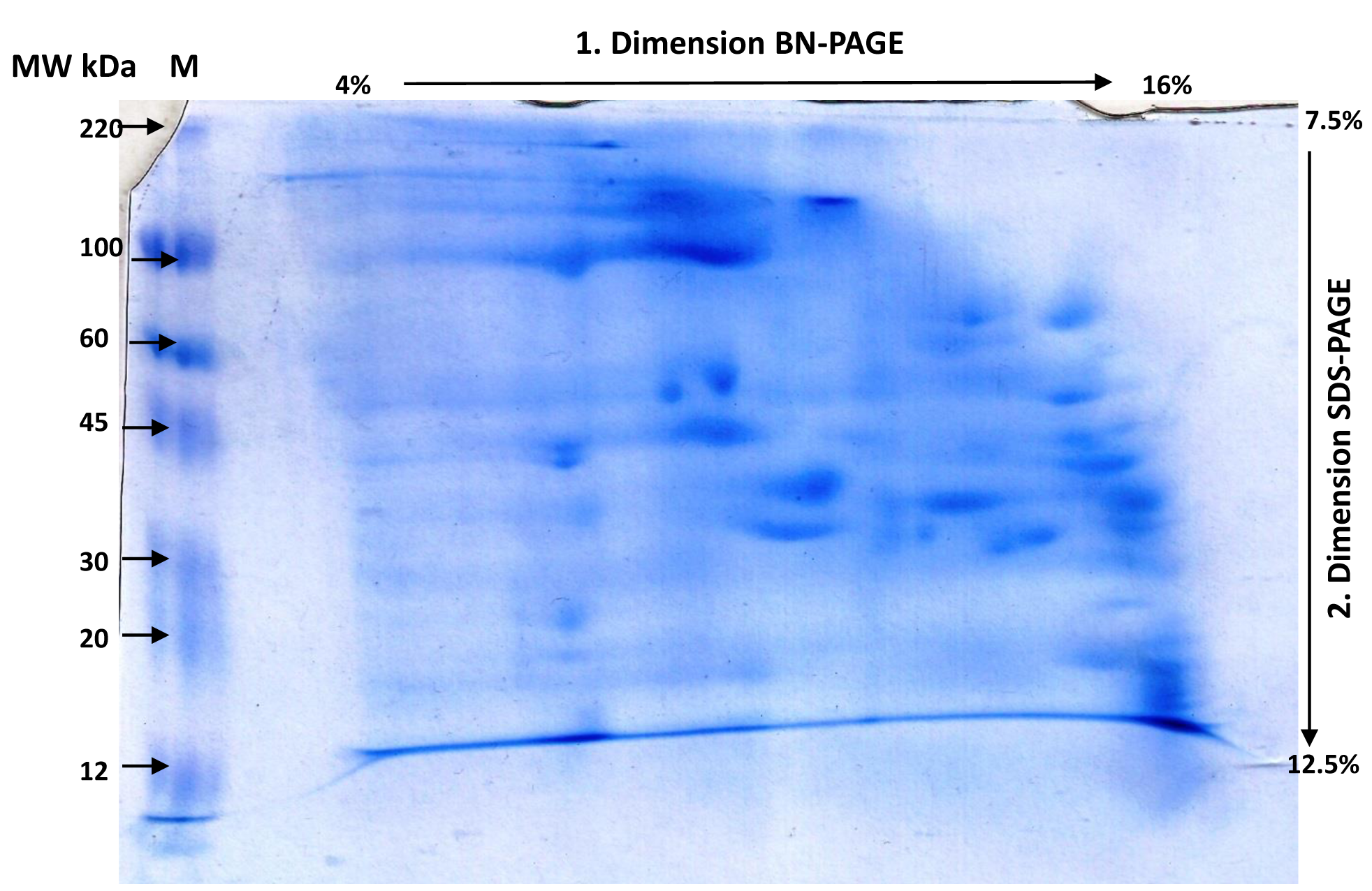

Supplement: Supplementary file 7 — Supplementary Figure S7. [file 41598_2021_93356_MOESM7_ESM.tif]

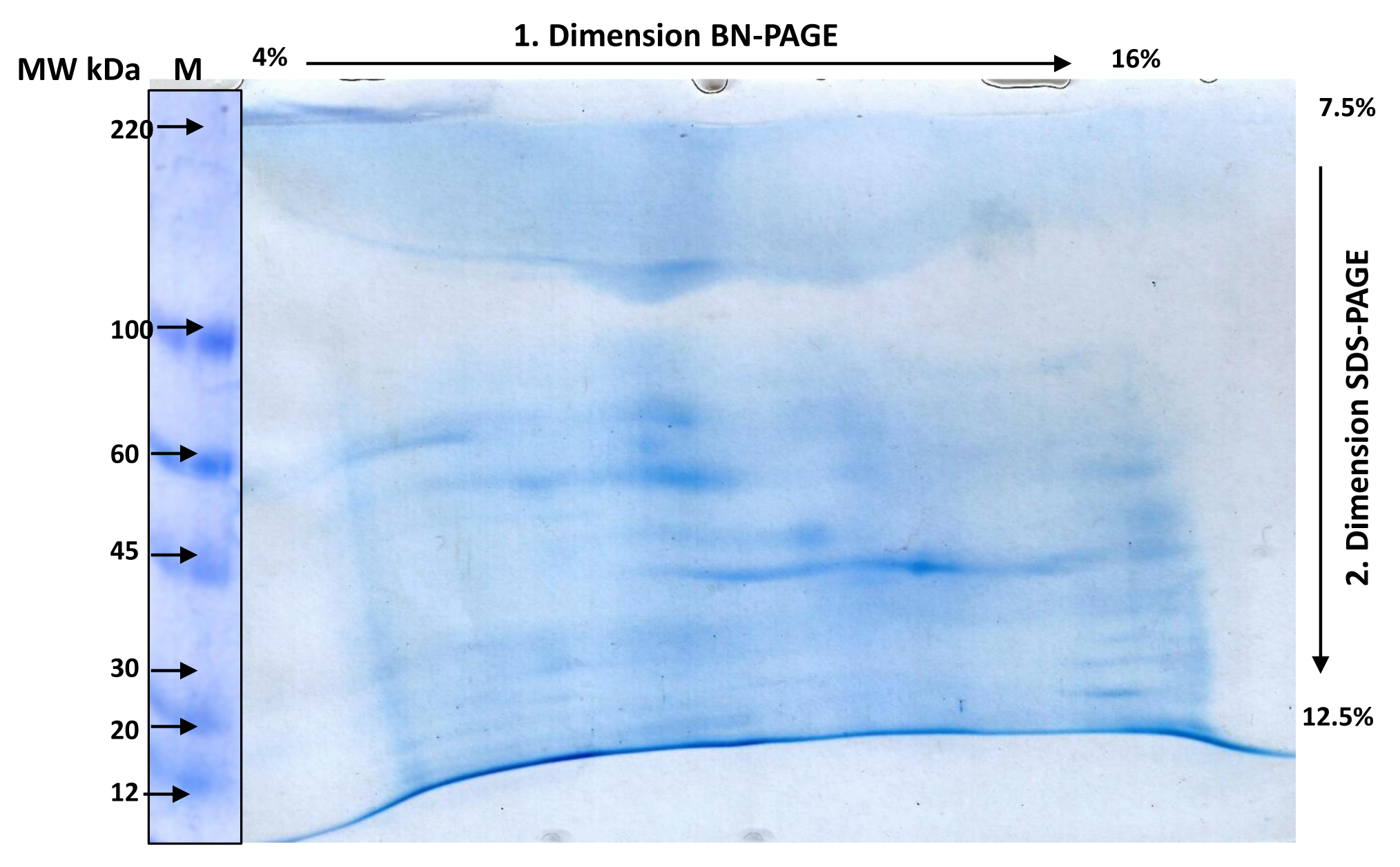

Supplement: Supplementary file 8 — Supplementary Figure S8. [file 41598_2021_93356_MOESM8_ESM.tif]

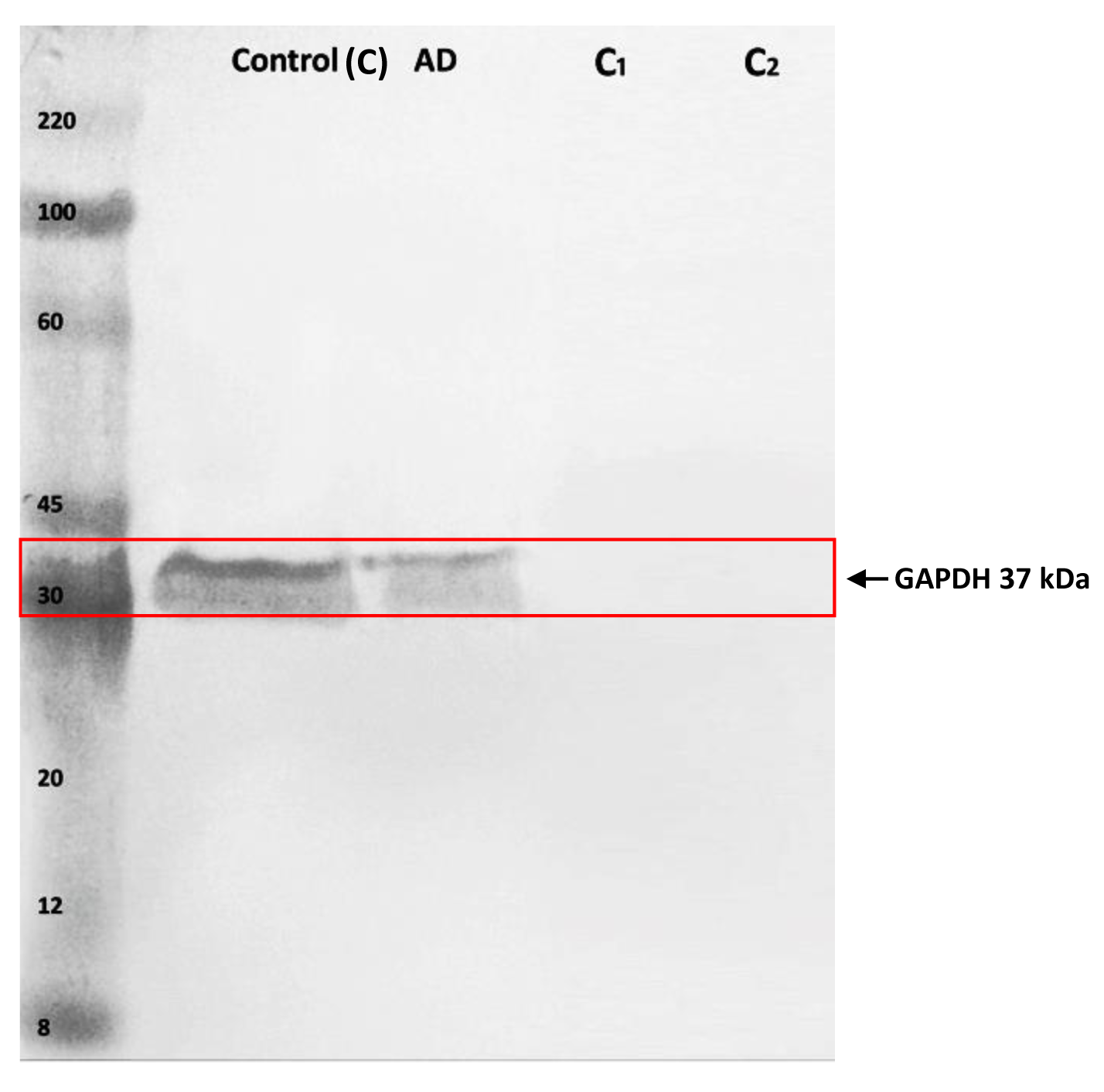

Supplement: Supplementary file 9 — Supplementary Figure S9. [file 41598_2021_93356_MOESM9_ESM.tif]

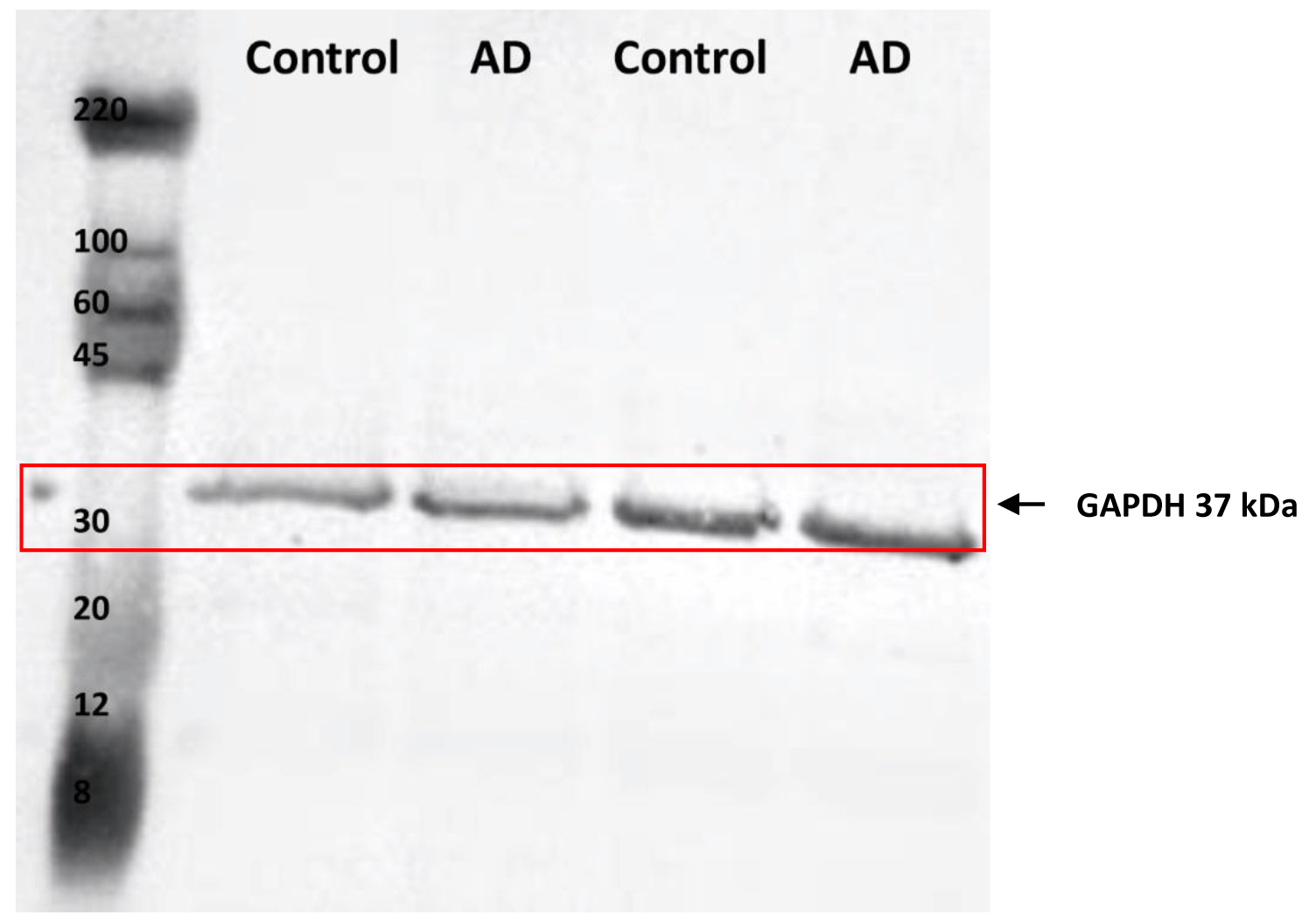

Supplement: Supplementary file 10 — Supplementary Figure S10. [file 41598_2021_93356_MOESM10_ESM.tif]
