# Supplementary material for: Characterization of functional protein complexes from Alzheimer’s disease and healthy brain by mass spectrometry-based proteome analysis
Source: Sci Rep. 2021 Jul 6;11:13891. doi: 10.1038/s41598-021-93356-9 (PMC8260596; doi:10.1038/s41598-021-93356-9)
Supplement: Supplementary file 11 — Supplementary Legends. [file 41598_2021_93356_MOESM11_ESM.pdf]

Supplementary Figure Legends

Fig S1. Pattern of protein complexes (I-XIII) obtained from the separation of control and AD sample resolved on 1D BN-PAGE 4-16% gel.
M: Molecular weight marker
C: Healthy control subjects
AD: Alzheimer's disease subjects

Fig S2. Pattern of protein complexes (I-XIII) obtained from the separation of control and AD sample resolved on 1D BN-PAGE 4-16% gel.
M: Molecular weight marker
C: Healthy control subjects
AD: Alzheimer's disease subjects

Fig S3. Pattern of protein complexes (I-XIII) obtained from the separation of control and AD sample resolved on 1D BN-PAGE 4-16% gel.
M: Molecular weight marker
C: Healthy control subjects
AD: Alzheimer's disease subjects

Fig S4. BN/SDS-PAGE map acquired from separation of ageing control protein complexes isolated from human brain prefrontal cortex. Representative spots separated on 12.5-7.5% SDS-PAGE in second dimension and Coomassie stained.
M: Molecular weight marker.

Fig S5. BN/SDS-PAGE map acquired from separation of ageing control protein complexes isolated from human brain prefrontal cortex. Representative spots separated on 12.5-7.5% SDS-PAGE in second dimension and Coomassie stained.
M: Molecular weight marker.

Fig S6. BN/SDS-PAGE map acquired from separation of ageing control protein complexes isolated from human brain prefrontal cortex. Representative spots separated on 12.5-7.5% SDS-PAGE in second dimension and Coomassie stained.
M: Molecular weight marker.

Fig S7. BN/SDS-PAGE map acquired from separation of AD protein complexes isolated from human brain prefrontal cortex. Representative spots separated on 12.5-7.5% SDS-PAGE in second dimension and Coomassie stained.
AD: Alzheimer's Disease
M: Molecular weight marker.

Fig S8. BN/SDS-PAGE map acquired from separation of AD protein complexes isolated from human brain prefrontal cortex. Representative spots separated on 12.5-7.5% SDS-PAGE in second dimension and Coomassie stained.
AD: Alzheimer's Disease
M: Molecular weight marker.

Fig S9. Co-immunoprecipitation of beta-actin with GAPDH for the identification of protein interaction in human brain prefrontal cortex. Human brain tissue proteins from AD patients and age matched control after solubilization in dodecyl-maltoside were collected using specific anti beta-actin antibody. The immunoprecipitaes were separated by SDS-PAGE electrophoresis followed by western blotting with anti GAPDH antibody. Immunoreactive band of GAPDH observed at 37 kDa. Two controls were included in the co-IP analysis: (C1) protein sepharose beads were incubated with extracted proteins in the absence of anti beta-actin antibody, (C2) protein sepharose beads were incubated with anti beta-actin antibody in the absence of extracted proteins.
AD: Alzheimer's Disease
C1: control 1
C2: control 2

Fig S10. Co-immunoprecipitation of beta-actin with GAPDH for the identification of protein interaction in human brain prefrontal cortex. Human brain tissue proteins from AD patients and age matched control after solubilization in dodecyl-maltoside were collected using specific anti beta-actin antibody. The immunoprecipitaes were separated by SDS-PAGE electrophoresis followed by western blotting with anti GAPDH antibody. Immunoreactive band of GAPDH observed at 37 kDa. Representative gel obtained from triplicate set of experiments.
AD: Alzheimer's Disease
