# Supplementary material for: Characterization of functional protein complexes from Alzheimer’s disease and healthy brain by mass spectrometry-based proteome analysis
Source: Sci Rep. 2021 Jul 6;11:13891. doi: 10.1038/s41598-021-93356-9 (PMC8260596; doi:10.1038/s41598-021-93356-9)
Supplement: Supplementary file 12 — Supplementary Table 1. [file 41598_2021_93356_MOESM12_ESM.pdf]

Supplementary table 1. Protein complexes and differentially expressed protein components in AD prefrontal cortex as identified by Nano LC-MS/MS.

Accession no. is obtained from SWISS/ Prot. Biological process and subcellular localization were determined by UniProt (www.uniprot.org) and PANTHER (http://www.pantherdb.org) database. The fold change was obtained from Melanie 7.0 (GeneBio) image analysis software generated statistical analysis and significance ($p<0.05$) was calculated from Student's *t*-test.

Accession No	Spot ID	Protein names	Gene symbols	Mr	Score	Peptide Matched	Sequences	Biological Process	Subcellular Location	Fold Change	p-value
						**Complex I**					
P02794	1	Ferritin heavy chain	FRIH	21.2	609	69(21)	10(7)	Iron storage	Autolysosome, cytosol, intracellular ferritin complex, mitochondrion, nucleus	2.4	0.0001
						**Complex II**					
Q9Y6R1	2	Electrogenic sodium bicarbonate cotransporter 1	SLC4A4	121.2	96	9(2)	7(2)	Ion transport	Basolateral cell membrane	-3.7	0.001
						**Complex III**					
Q93050	3	V type proton ATPase 116 KDa	ATP6V0A1	96.3	896	51(22)	28(15)	Ion transport	Cytoplasmic vesicle membrane, Melanosome.	2.2	0.0001
P43004	4	Excitatory aminoacid transporter 2	SLC1A2	62.1	129	5(3)	5(3)	Amino acid transport	Cell membrane	2.4	0.0001
P31930	5	Cytochrome b-c1 complex subunit 1	UQCRC1	52.6	76	18(8)	11(2)	Electron transport chain	Mitochondrion inner membrane	1.4	0.0001
P22695	6	Cytochrome bc1complex subunit 2	UQCRC2	48.4	361	20(7)	14(4)	Electron transport chain	Mitochondrion membrane	1.3	0.0095
P08574	7	Cytochrome c1 heme protein	CYC1	35.3	127	6(2)	5(2)	Electron transport chain	Mitochondrion membrane	1.2	0.0001
Q16653	8	Myelin oligodendrocyte glycoprotein	MOG	28.2	313	11(8)	7(6)	Cell adhesion	Cell membrane	1.1	0.9242

					**Complex IV**						
P10620	9	Microsomal glutathione S transferase	MGST1	17.5	83	1(1)	1(1)	Oxidation reduction process	Mitochondrion membrane, ER and Microsome membrane	-5.7	0.031
Q969P0	10	Immunoglobulin super family member 8	IGSF8	64.9	61	5(1)	3(1)	Nervous system development, cell proliferation.	Cell membrane	1.5	0.0127
					**Complex V**						
P12814	11	Alpha-actinin-1	ACTN1	102.9	625	41(19)	27(17)	Actin bundling, Apoptosis regulation	Cell membrane, cytoskeleton, Z line, cell junction	-3.1	.0001
P07900	12	heat shock protein 90 alpha	HSP90AA1	84.67	88	7(2)	7(2)	Stress response	Cytoplasm. Melanosome. Cell membrane	1.6	0.046
					**Complex VI**						
P14618	13	Pyruvate kinase	KPYM	57.9	143	10(4)	10(4)	Glycolysis	Cytoplasm. Nucleus	1.1	0.1
Q9Y2T3	14	Guanine deaminase	GDA	50.9	99	3(2)	2(1)	Guanine metabolism, nervous system development	Cytosol	-1.2	0.0077
P07195	15	L-lactate dehydrogenase B chain	LDHB	36.6	355	14(8)	9(7)	Oxidation-reduction	Cytoplasm	2.9	0.0177
					**Complex VII**						
P13798	16	Acyl aminoacid releasing enzyme	APEH	81.1	80	2(1)	2(1)	Proteolysis, amyloid beta metabolic process	Cytoplasm	2.5	0.0061
P14136	17	Glial fibrillary acidic protein	GFAP	49.8	229	8(1)	13(8)	Cytoskeleton organization, Chaperone mediated autophagy	Cytoplasm	-1.3	0.0053
P04406	18	Glyceraldehyde-3-PO_4DH	GAPDH	36.03	201	12(4)	7(2)	Apoptosis, Glycolysis, Translation regulation	Cytoplasm, Cytoskeleton, Membrane, Nucleus	1.9	0.0087
P60201	19	Myelin proteolipid protein	PLP1	30.07	199	11(4)	7(3)	Myelination	Cell membrane, myelin membrane	2.2	0.0276
P46821	21	Microtubule associated protein 1B	MAP1B	27.04	56	3(2)	2(2)	Microtubule bundling	Cytoplasm, cytoskeleton, cell junction, synapse, dendritic spine	-3.5	0.01
P60709	20	Actin cytoplasmic	ACTB	41.7	187	10(6)	9(6)	Subcellular components trafficking, membrane organization	Cytoskeleton	5.1	0.0094
P00403	22	Cytochrome c oxidase subunit 2	COX2	25.5	72	4|(2)	2(2)	Electron transport chain	Mitochondrion inner membrane	NS	0.049

					**Complex VIII**						
P61764	23	Syntaxin-binding protein 1	STXBP1	67.5	431	27(10)	21(9)	Protein transport, transport	Cytoplasm, Peripheral membrane protein	3.6	0.0001
P08238	24	Heat shock protein 90B	HS90B	83.2	203	10(4)	9(4)	Stress response	Cytoplasm, nucleus, cell membrane, melanosome	1.2	0.025
P13637	25	Sodium potassium transporting ATPAse	AT1A3	112.8	404	23(12)	20(12)	Ion transport	Cell membrane	1.1	0.096
					**Complex IX**						
P49588	26	Alanine--tRNA ligase,	SYAC	106.7	197	11(5)	10(5)	Protein biosynthesis	Cytoplasm	1.4	0.9
Q99798	27	Aconitate Hydratase	ACO2	85.3	105	4(1)	4(1)	Tricarboxylic acid cycle	Mitochondria	4.0	0.0011
P16435	28	NADPH--cytochrome P450 reductase	POR	96.6	81	2(2)	2(2)	Oxidation-reduction process	Endoplasmic reticulum membrane	1.3	0.1035
					**Complex X**						
P25705	29	ATP synthase subunit alpha, mitochondrial	ATP5A1	59. 7	326	23(10)	18(8)	ATP synthesis, ion transport	Mitochondrion membrane, cell membrane	2.2	0.0077
P06576	30	ATP synthase subunit beta, mitochondrial	ATP5B	56.5	281	23(9)	13(8)	ATP synthesis, ion transport	Mitochondrion membrane	2.3	0.0029
					**Complex XI**						
P17600	31	Synapsin-1	SYN1	74.1	221	5(5)	4(4)	Neurotransmitter secretion	Golgi apparatus, synapse	1.4	0.95
					**Complex XII**						
P09104	32	Gamma-enolase	ENO2	47.2	134	4(3)	4(3)	Glycolysis	Cytoplasm, Cell Membrane	-1.2	0.87
					**Complex XIII**						
Q16623	33	Syntaxin-1A	STX1A	33.03	132	5(2)	5(2)	Exocytosis	Cell membrane, synaptic membrane	1.2	0.0001
O94811	34	Tubulin polymerization-promoting protein	TPPP	23.67	99	5(3)	3(2)	Tubulin polymerization	Nucleus, cytoskeleton, cytoplasm	-3.72	0.0061
